# Urethral Foreign Body Management: A Case Report

**DOI:** 10.1100/tsw.2004.47

**Published:** 2004-06-07

**Authors:** Andy Y. Chang, Chester J. Koh, John P. Stein

**Affiliations:** Department of Urology, University of Southern California, Keck School of Medicine, Los Angeles, CA, USA

## Abstract

The management of urethral foreign bodies may require the use of various surgical techniques in a urologist's armamentarium. We report a unique case of a urethral foreign body requiring percutaneous and endoscopic techniques for removal.

## INTRODUCTION

The management of urethral foreign bodies may require the use of various surgical techniques. A review of the literature revealed multiple methods for their extraction. We report a case of a urethral foreign body, a ball-point pen, traversing the prostatic urethra and lodging in the bulbar urethra. The foreign body was removed using the combination of a small perineal incision and cystoscopy, avoiding the need for general anesthesia. To our knowledge, the combined use of percutaneous and endoscopic techniques for urethral foreign body management has not previously been reported.

## CASE REPORT

A 40-year-old male presented to the emergency room with a one-month history of perineal pain and hematuria after physical activity. The patient denied any obstructive symptoms. Physical examination was significant for a palpable foreign body in the midline perineum inferior to the scrotal border. On digital rectal exam, the foreign body was palpable along the anterior rectal wall. A plain radiograph of the abdomen and pelvis confirmed the presence of a thin foreign body spanning the bulbar urethra and the bladder neck (Figure 1). Cystoscopy confirmed the presence of a ball-point pen, which had eroded through the ventral surface of the bulbar urethral wall. The foreign body did not appear amenable to removal via an endoscopic approach. Using local anesthesia, a small one-centimeter incision was made along the perineal raphe down to the foreign body. This incision facilitated the removal of the inner ink cartridge; however, the pen's plastic outer sheath dislodged from the ink cartridge and remained in the urethra. The outer sheath was subsequently removed with rigid cystoscopic graspers and the foreign body was identified as a ball-point pen. A 22 French Councill tip catheter was placed over a guide wire and the perineal incision was closed in two layers with absorbable sutures. The Councill tip catheter was left

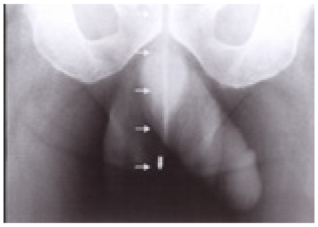

**FIGURE 1.** Abdominal-pelvic radiograph with the foreign body spanning the bulbar urethra, prostatic urethra, and the bladder neck. Arrows denote the location of the pen.

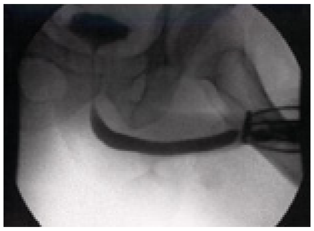

**FIGURE 2**. Retrograde urethrogram obtained after the Councill tip catheter was removed. The Councill tip catheter was left indwelling for 3 weeks after removal of the foreign body and repair of the urethra.

indwelling for 3 weeks. A retrograde urethrogram (RUG) obtained after catheter removal revealed no evidence of extravasation or stricture (Figure 2). Two years after the foreign body removal, the patient still reports no voiding difficulties.

## CONCLUSION

Several cases of urethral foreign bodies have been described in the literature. (1-3) The reasons for urethral insertion are multifold, ranging from dementia (2) to intoxication (1) to sexual experimentation and/or play. (3) Regardless of the motive for placement of these foreign bodies, their extrications can be challenging and may require the creative use of urologists' surgical armament in their removal. In this reported case, a minimally invasive technique was employed to remove the urethral foreign body, avoiding the use of general anesthesia. The combination of a small perineal incision and cystoscopy was successfully used for this potentially challenging clinical dilemma. In conclusion, the techniques of urethral foreign body removal should be as varied as the foreign bodies themselves and should be dictated by the needs of the individual case. To our knowledge, the combination of both percutaneous and endoscopic techniques in the removal of a urethral foreign body has not been previously reported.

## References

1. Van Ophoven A, De Kernion JB: Clinical management of foreign bodies of the genitourinary tract. J Urol, 164(2):274-87, 2000.
2. Phillips J: Fogarty catheter extraction of unusual urethral foreign bodies. J Urol, 155(4):1374-5, 1996.
3. Ghaly AFF, Munishankar AR, Sultana SR, Nimmo M: Case report: foreign body in male penile urethra. Genitourin Med, 72(1):67-8, 1996.
